# Increased Levels of Brain-Derived Neurotrophic Factor Are Associated With High Intrinsic Religiosity Among Depressed Inpatients

**DOI:** 10.3389/fpsyt.2019.00671

**Published:** 2019-09-13

**Authors:** Bruno Paz Mosqueiro, Marcelo P. Fleck, Neusa Sica da Rocha

**Affiliations:** ^1^Programa de Pós-graduação em Psiquiatria e Ciências do Comportamento, Universidade Federal do Rio Grande do Sul (UFRGS), Porto Alegre, Brazil; ^2^Hospital de Clínicas de Porto Alegre (HCPA), Porto Alegre, Brazil; ^3^Interventions and Innovations for Quality of Life (I-QOL), Universidade Federal do Rio Grande do Sul (UFRGS), Porto Alegre, Brazil

**Keywords:** depression, brain-derived neurotrophic factor, neuroplasticity, religion, spirituality

## Abstract

Recognition of the importance of religion and spirituality in psychiatry is increasing, and several studies have shown a predominantly inverse relationship between religiosity and depression. Brain-derived neurotrophic factor (BDNF) is a widely studied brain neurotrophin responsible for synaptic plasticity, dendritic and neuronal fiber growth, and neuronal survival. The objective of the present study was to evaluate BDNF levels across high and low intrinsic religiosity (IR) in depressed inpatients. Serum BDNF levels were evaluated from 101 depressed inpatients at hospital admission and 91 inpatients at discharge. Religiosity was assessed using a validated version of the Duke University Religion Index. High IR patients had significantly higher serum BDNF at discharge than do low IR (52.0 vs. 41.3 ng/mL, P = 0.02), with a Cohen’s d effect size difference of 0.56. High IR patients had a statistically significant increase in BDNF levels from admission to discharge (43.6 ± 22.4 vs. 53.8 ± 20.6 ng/mL, −1.950 (paired t-statistic), P = 0.05). The relationship between IR and BDNF levels (F = 6.199, P = 0.00) was controlled for the effects of depressive symptoms ( *β* = 2.73, P = 0.00) and psychiatric treatments, including selective serotonin reuptake inhibitors (SSRIs) (*β* = 0.17, P = 0.08), serotonin and norepinephrine reuptake inhibitors (SNRIs) ( *β* = −0.23, P = 0.02), tricyclic antidepressants (TCAs) ( *β* = −0.17, P = 0.10), lithium ( *β* = 0.29, P = 0.00), anticonvulsants ( *β* = 0.22, P = 0.03), antipsychotics ( *β* = −0.05, P = 0.61), and electroconvulsive therapy ( *β* = 0.00, P = 0.98). The current findings suggest a potential pathway to help understand the protective effect of religiosity in depressive disorders.

## Introduction

Over past decades, religion and spirituality have become increasingly important topics in psychiatry research (1). Although epidemiological studies in this context have provided evidence for an inverse relationship between religiosity and depression (2), there is limited empirical data on biological correlates that could mediate their relationship (3).

Brain-derived neurotrophic factor (BDNF) is one of the most widely studied brain neurotrophins (4). BDNF influences synaptic plasticity and dendritic and neuronal fiber growth, promotes neuronal survival (4), and has been proposed as a biological marker of brain neuroplasticity (5). Meta-analyses have shown that BDNF concentrations are lower in patients with depression than in healthy controls and that levels increase with successful antidepressant treatment and/or recovery from depression (6).

Previous studies have reported an association between religious attendance and decreased levels of interleukin-6, a pro-inflammatory cytokine linked to higher mortality in older adults as well as depression (7, 8). Other studies have shown that individuals declaring a higher importance for religion or spirituality had a 90% decreased risk of depression and higher cortical thickness (3, 9). Furthermore, higher intrinsic religiosity (IR) has been associated with resilience, quality of life, greater improvement of depressive symptoms, and fewer suicide attempts in a sample of depressed inpatients (10). Nonetheless, to the best of our knowledge, no previous study has yet evaluated whether religiosity correlates with BDNF levels, especially in depressed subjects. Therefore, the aim of the present study was to evaluate whether serum BDNF levels correlate with IR in a sample of depressed inpatients.

## Materials and Methods

### Patient Sample

The present study evaluated a subset of patients in a prospective cohort study at the Hospital de Clínicas de Porto Alegre, a university hospital and tertiary psychiatric care referral center in southern Brazil. From 634 subjects admitted to inpatient psychiatric care from May 2011 to December 2013, 196 presented with depressive episodes. Of them, serum BDNF levels and complete protocol assessments were consecutively evaluated from 101 depressed inpatients at hospital admission and 91 inpatients at discharge. Written informed consent was obtained from all patients prior to study inclusion according to approval provided by the hospital ethical committee. Patients with clinical comorbidities that could interfere with BDNF analysis (acute or chronic infections, autoimmune or endocrine diseases, neoplasia, etc.), significant cognitive deficits that limited comprehension of self-report instruments, drug or alcohol addiction or dependence as a main diagnostic, and/or current hypomanic or manic episodes were excluded from the present study.

### Assessments

Diagnosis of psychiatric disorders was performed using the Brazilian Portuguese version of the Mini International Neuropsychiatric Interview. All subjects meeting criteria for a depressive episode underwent a comprehensive evaluation, including a general protocol with clinical and sociodemographic information and Brazilian Portuguese-validated versions of the Hamilton Depression Rating Scale (HAM-D), Cumulative Illness Rating Scale (CIRS), General Assessment of Functioning (GAF) Scale, Clinical Global Impression (CGI) Scale, and Brief Psychiatric Rating Scale (BPRS). Resilience scores were evaluated with the Brazilian-validated version of the Resilience Scale, a 25-item Likert scale tool with scores ranging from 25 to 175 (higher scores indicate greater resilience). All assessments were performed within the first 72 h of hospital admission and within the 48 h before hospital discharge.

### Religiosity

Religiosity was assessed using the Brazilian Portuguese-validated version of the Duke University Religion Index (DUREL). The DUREL is a 5-item Likert scale assessment tool with three dimensions of religiosity. The first question evaluates the dimension of organizational religiosity (e.g., church, temple, or institutional attendance), and the second evaluates nonorganizational religiosity (individual religious activities performed in private, such as prayer, religious readings, and meditation). The last three questions comprise the IR dimension, the level of religious commitment, and how much religiousness represents a central part of everyday life as a source of belief, motivation, and meaning. IR was chosen as the main dimension of religiosity to predict clinical outcomes in depressed inpatients. Religiosity was assessed at time of hospital discharge, in order to avoid influences of acute psychiatric symptoms at time of admission. Individuals presenting a total score of more than 10 points on the last three questions of the DUREL combined were further categorized into high and low IR groups (11).

### BDNF Levels

Serum samples were collected within the first 72 h of hospital admission and within the 48 h before discharge. The laboratory research assistants who collected and analyzed BDNF serum samples were blinded to clinical and religious measures of depressed patients. Blood samples (10 mL) were drawn by venipuncture into an anticoagulant-free vacuum tube and then centrifuged at 4,000 × *g* for 10 min. Serum was stored at −80°C until analysis. BDNF levels in all samples were analyzed by sandwich enzyme-linked immunosorbent assay (ELISA) using the same commercial kit (EMD Millipore Corporation, Billerica, MA, USA). All samples from all patients were analyzed using the commercial kit on the same date. Serum samples in sample diluent (1:100) were incubated on 96-well microtiter plates (flat-bottom), along with BDNF standards (7.8–500 pg of BDNF), for 24 h at 4°C. Plates were then washed four times with wash buffer followed by incubation with a biotinylated mouse antihuman BDNF monoclonal antibody (1:1,000 in sample diluent) at room temperature for 3 h. Plates were washed again four times with wash buffer and then incubated with a streptavidin–horseradish peroxidase conjugate solution (1:1,000 in sample diluent) at room temperature for 1 h. After addition of substrate and stop solution, BDNF content was determined by measuring the absorbance of each sample at 450 nm. The standard curve demonstrates a direct relationship between optical density and BDNF concentration. All BDNF results are expressed in ng/mL.

### Statistical Analysis

A Kolmogorov–Smirnov (KS) test was applied to assess the normality of the sample distribution. The KS test indicated that BDNF serum levels at admission (0.89, *P* = 0.40) and discharge (0.72, *P* = 0.67) were normally distributed. First, a Pearson’s correlation analysis was performed to evaluate the correlation between scores of IR and BDNF serum levels at admission and discharge. A one-tailed analysis was conducted to test for the hypothesis of a positive association between variables. Second, depressed inpatients were categorized into high and low IR groups. A paired *t*-test analysis was carried out to determine whether the differences between mean BDNF serum levels from admission to discharge were different across high and low IR groups of depressed inpatients. In the next step, a multivariate analysis of variance was conducted in order to test for the effects of IR on BDNF samples analyzed on the same time. BDNF levels at admission and discharge were tested as related dependent variables. High and low IR groups (high IR = 1 vs. low IR = 0) were analyzed as a categorical independent variable, and the variables age, sex, depressive symptoms at admission, depressive symptoms at discharge, resilience, and tobacco consumption were tested as covariates. A complementary linear regression analysis evaluated the relationship between IR (high IR = 1 vs. low IR = 0) and BDNF serum levels at discharge of psychiatric unit, to control for the effects of psychiatric treatments, including selective serotonin reuptake inhibitors (SSRIs), serotonin and norepinephrine reuptake inhibitors, tricyclic antidepressants (TCAs), lithium, anticonvulsants, antipsychotics, and electroconvulsive therapy (ECT). Statistical analyses were performed using SPSS version 20 software. Data are presented as means ± standard deviations or percentages unless specified otherwise.

## Results

### Sociodemographic and Clinical Characteristics

Among the depressed inpatients, 59.3% were female, 92.3% were white, and the mean age was 46.2 years. Most individuals presented with severe symptomatology in HAM-D, CGI, and BPRS assessments and reported a mean of 2.8 previous psychiatric admissions. There were no statistically significant differences between low and high IR groups across sociodemographic or clinical characteristics, including age, sex, ethnicity, marital status, education, socioeconomic level, number of previous psychiatric hospitalizations, ECT treatment, length of inpatient treatment, prior or current illicit substance use, previous suicide attempts, current tobacco smoking, and clinical scores regarding depressive symptoms (HAM-D), general psychopathology (CGI and BPRS), and functionality (GAF), at admission or discharge (**Table 1**). High IR patients were more likely to be using SSRI antidepressants at discharge than were low IR patients (51.4% vs. 29.6%, *P* = 0.05).

**Table 1:** Sociodemographic and clinical variables in low and high intrinsic religious depressed inpatients (*n* = 101).

(%)	Low IR	High IR	Statist*	*P*-value
Female	33.3%	44.6%	1.033	0.36
Ethnicity White Non-white	92.6% 7.4%	81.9% 18.1%	1.73	0.22
Marital Status Single Married Separated Widowed	34.6% 34.6% 23.1% 7.7%	31.5% 45.2% 20.5% 2.7%	1.78	0.61
Education, years of study				
Occupation Student Employed Unemployed Stay at home Health Insurance Retired Prefer not to mention	3.8% 26.9% 15.4% 7.7% 26.9% 11.5% 7.7%	1.4% 30.6% 22.2% 4.2% 26.4% 8.3% 6.9%	1.78	0.93
Performed ECT (yes/no)	44.4%	27.0%	2.77	0.09
Illicit substance use lifetime (yes/no)	22.2%	30.4%	0.44	0.50
Tobacco consumption (yes/no)	45.5%	34.6%	0.77	0.43
Suicide attempts (yes/no)	66.7%	64.4%	0.04	0.83
Previous mania	33.3%	33.8%	0.00	0.96
Previous hypomania	14.8%	8.2%	0.95	0.32
SSRI discharge	29.6%	51.4%	3.76	0.05**
TCA discharge	3.7%	6.8%	0.33	0.56
SNRI discharge	11.1%	5.4%	0.99	0.31
Mood stabilizers	22.2%	29.7%	0.55	0.45
Antipsychotics	70.4%	63.5%	0.41	0.52
**(Means)**	**Low IR**	**High IR**	Statist**	***P*-value**
Age	46.5	45.2	0.41 (t)	0.68
Number of psychiatry hospital admissions	2.59	2.99	982.0 (M)	0.97
Length of inpatient care in days	27.5	30.8	962.0 (M)	0.77
Number of lifetime suicide attempts	2.04	1.80	634.0 (M)	0.77
CIRS global scores	1.21	1.38	−1.50 (t)	0.13
HAM-D admission	22.6	23.2	−0.39 (t)	0.69
HAM-D discharge	8.32	7.26	0.89 (t)	0.37
BPRS admission	21.5	24.4	−1.23 (t)	0.22
BPRS discharge	9.73	9.02	0.39 (t)	0.69
CGI admission	5.00	5.35	659.5 (M)	0.11
CGI discharge	3.38	3.23	575.5 (M)	0.11
Resilience (RS)	112.0	133.1	−3.10 (t)	0.00***

*Chi-square tests for categorical variables, t-test (t) and Mann–Whitney (M) tests for mean values. **Statistically significant values in bold for P < 0.05. ***Statistically significant values in bold for P < 0.01.

BPRS, Brief Psychiatric Rating Scale; CGI, Clinical Global Impression; CIRS, Cumulative Illness Rating Scale; ECT, electroconvulsive therapy; HAM-D, Hamilton Depression Rating Scale; SNRI, serotonin and norepinephrine reuptake inhibitors; SSRI, selective serotonin reuptake inhibitor; TCA, tricyclic antidepressant.

### Religiosity and BDNF Levels

**Figure 1:**
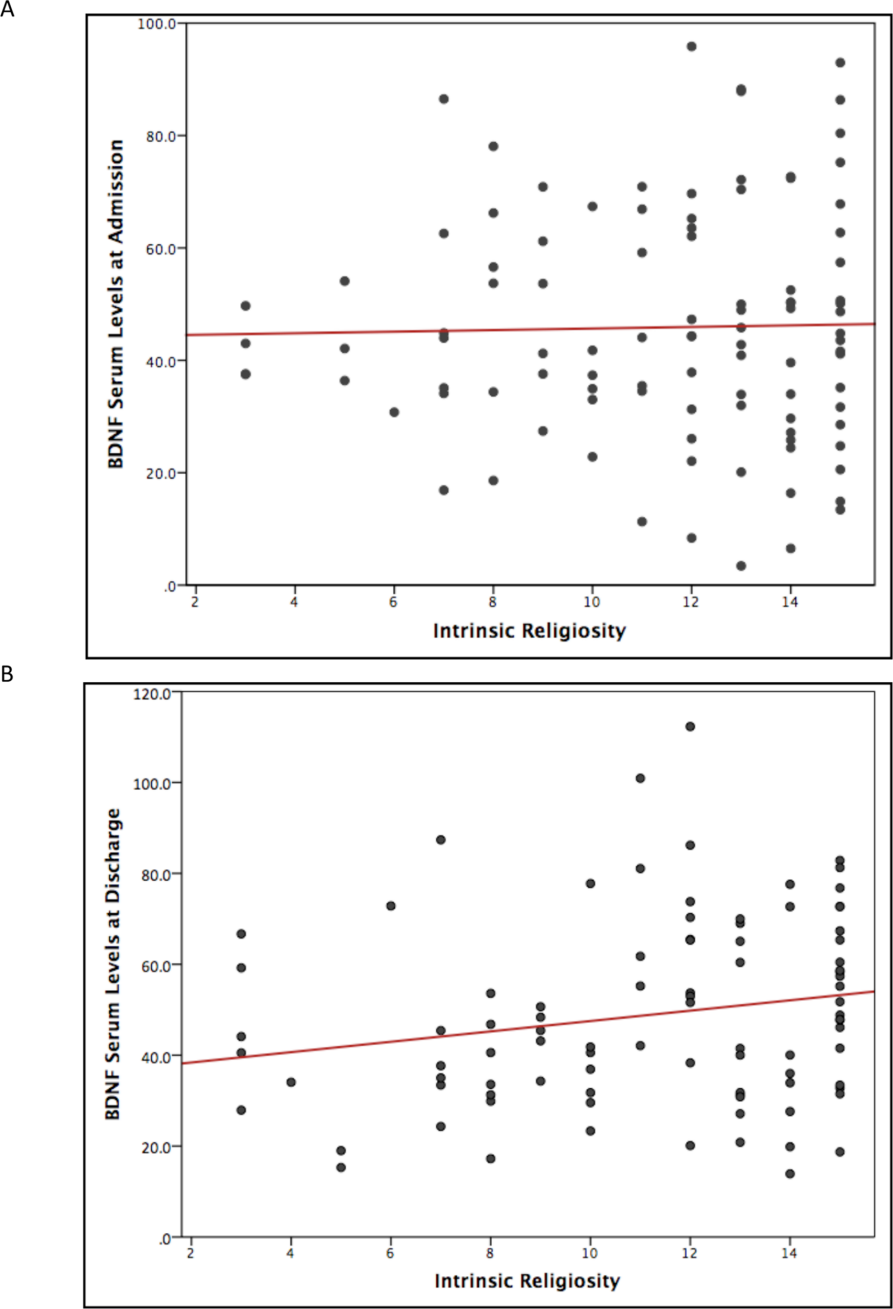
Scatter plot of correlations between intrinsic religiosity and BDNF serum levels of depressed inpatients. **(A)** Pearson’s correlation coefficient scatter plot of BDNF serum levels (ng/mL) at time of hospital admission (*n* = 101, *r* = 0.02, *P* = 0.41). **(B)** Pearson’s correlation coefficients of BDNF serum levels (ng/mL) at time of hospital discharge (n = 91, r = 0.19, P = 0.03). BDNF, brain-derived neurotrophic factor; IR, intrinsic religiosity.

A statistically significant correlation between IR and BDNF serum levels was identified at hospital discharge (*n* = 91, *r* = 0.19, one-tailed, *P* = 0.03, **Figure 1**). The correlation was not statically significant between IR and BDNF serum levels at admission (*n* = 101, *r* = 0.02, one-tailed, *P* = 0.41, **Figure 1**).

**Figure 2:**
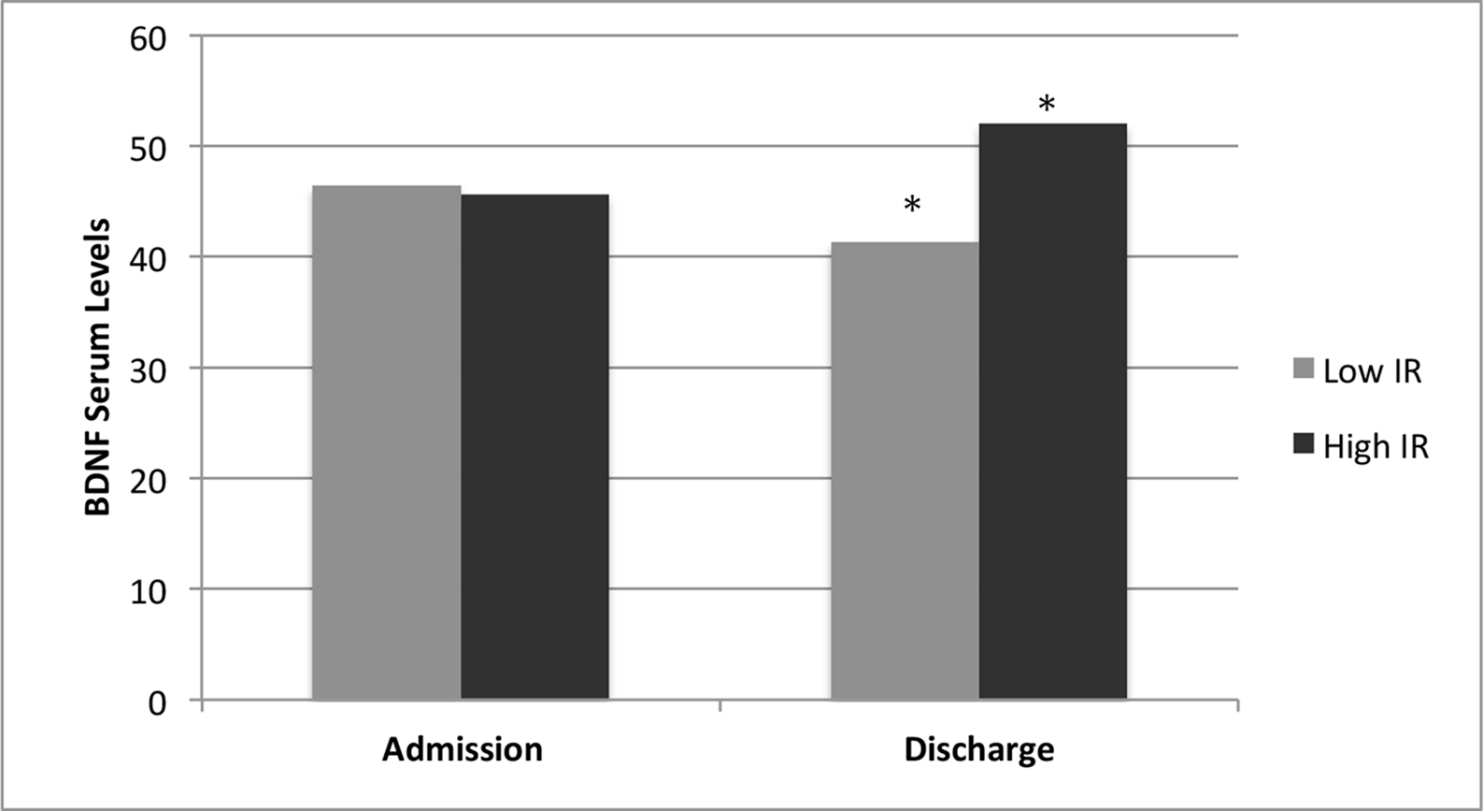
Intrinsic religiosity and BDNF serum levels of Depressed Inpatients. BDNF serum levels (ng/ML) at time ofhospital admission (46.4 vs. 45.6, *P* = 0.85, *n* = 101) and discharge (41.3 vs. 52.0, *P* = 0.02, *n* = 91) in low and high intrinsic religiosity (IR) depressed Inpatients. *Statistically significant difference between groups (*P* < 0.05). BDNF, brain-derived neurotrophic factor.

Compared categorically with IR groups, high IR patients had significantly higher mean serum BDNF levels at discharge than do IR patients (52.0 ± 21.3 vs. 41.3 ± 16.6 ng/mL, 2.314 (*t*-statistic), *P* = 0.02). Further analysis showed a moderate difference in serum BDNF levels between the IR groups, with a Cohen’s *d* effect size difference of 0.56 (**Figure 2**). On the other hand, no statistically significant differences in serum BDNF levels were found between low and high IR patients at hospital admission (46.4 ± 16.9 vs. 45.6 ± 21.7 ng/mL, 0.173 (*t*-statistic), *P* = 0.85). Paired *t*-test analysis showed that high IR patients had a statistically significant mean increase in BDNF levels from admission to discharge (43.6 ± 22.4 to 53.8 ± 20.6 ng/mL, −1.950 (*t*-statistic), *P* = 0.05, **Table 2**). On the other hand, no statistically significant differences in BDNF levels were found between admission and discharge in low IR patients (47.6 ± 15.9 to 43.6 ± 19.6 ng/mL, 0.84 (*t*-statistic), *P* = 0.40, **Table 2**).

**Table 2:** Paired *t*-test analysis of serum BDNF levels (ng/mL) across high and low Intrinsic religiosity groups of depressed inpatients.

Intrinsic religiosity (*t*-test)	Admission (*n* = 101)	Discharge (*n* = 91)	Statist	*P*-value
Low IR	46.4 (16.9)	41.3 (16.6)	0.173	0.85
High IR	45.6 (21.7)	52.0 (21.3)	2.314	**0.02**
**Intrinsic religiosity (paired *t*-test)**	**Admission**	**Discharge**		
Low IR (*n* = 29)	47.6 (15.9)*	43.6 (19.6)	0.84	0.40
High IR (*n* = 60)	46.3 (22.4)	53.8 (20.6)	−1.950	**0.05**

*Mean values and standard deviation ( ± SD). *Statistically significant values in bold for P < 0.05.

BDNF, brain-derived neurotrophic factor.

A multivariate analysis of variance was performed to test for the interactions between BDNF samples across low and high IR groups at same time, controlling for covariates (**Table 3**). The results showed a significant effect of the independent categorical variable IR on BDNF levels (Wilks’ lambda 0.754, *F*(6.199), *P* < 0.01). The effects were significant controlling for age (Wilks’ lambda 0.963, *F*(0.696), *P* = 0.50), sex (Wilks’ lambda 0.858, *F*(2975), *P* = 0.06), resilience (Wilks’ lambda 0.832, *F*(3.646), *P* = 0.03), depressive symptoms at admission (Wilks’ lambda 0.982, *F*(0.324), *P* = 0.72), depressive symptoms at discharge (Wilks’ lambda 0.865, *F*(2.816), *P* = 0.06), and tobacco consumption (Wilks’ lambda 0.854, *F*(3.073), *P* = 0.05). Univariate testing (between-subjects effects) indicated that the effect of IR was statistically significant at BDNF levels at discharge (*F* = 12.044, *P* = 0.001).

**Table 3:** Multivariate tests of BDNF serum levels (ng/mL) and intrinsic religiosity in depressed inpatients.

Independent variable	Wilks’ lambda	*F*	Sig.
Intrinsic religiosity (high = 1 vs. low = 0)	0.754	6.199	0.005**
**Covariates**			
Age	0.963	0.696	0.50
Sex	0.858	2.975	0.06
Resilience scale (RS)	0.832	3.646	0.03*
Depressive symptoms admission (HAM-D)	0.982	0.324	0.72
Depressive symptoms discharge (HAM-D)	0.865	2.816	0.07
Tobacco consumption	0.854	3.073	0.05*

Multivariate test of BDNF serum levels at time of admission and discharge of depressed inpatients (MANCOVA) | 1. Dependent variables: BDNF serum levels at admission and BDNF serum levels at discharge. 2. Independent variable: high and low intrinsic religiosity (high = 1). 3. Covariates: age, sex, resilience scale, HAM-D at admission, HAM-D at discharge, tobacco consumption (yes = 1). *Significant for P < 0.05; **Significant for a P < 0.01.

BDNF, brain-derived neurotrophic factor; HAM-D, Hamilton Depression Rating Scale; MANCOVA, multivariate analysis of covariance.

A complementary linear regression model evaluated the effect of psychiatric inpatient treatment at discharge (*n* = 82, *R* ^2^ = 0.32, adjusted *R* ^2^ = 0.24, *P* = 0.001, **Table 4**). In this model, IR remained a statistically significant independent variable (*β* = 0.26, *P* = 0.01) in relationship to serum BDNF levels at hospital discharge, after controlling for SSRI (*β* = 0.17, *P* = 0.08), serotonin and norepinephrine reuptake inhibitor (*β* = −0.23, *P* = 0.02), tricyclic antidepressants (*β* = −0.17, *P* = 0.10), lithium (*β* = 0.29, *P* = 0.00), anticonvulsants (*β* = 0.22, *P* = 0.03), antipsychotics (*β* = −0.05, *P* = 0.61), and ECT (*β* = 0.00, *P* = 0.98).

**Table 4:** Multilinear regression of BDNF serum levels (ng/mL) at hospital discharge in depressed inpatients controlling for psychiatric treatments (*n* = 82).

	Unstandardized Coefficients	Standardized Coefficients	T	Sig.	Colinearity statistics	
	B	B			Tolerance	VIF
Intrinsic religiosity (low/high)	11,949	0.26	2.635	**0.01**	0.919	1.088
SSRI	7,248	0.17	1.731	0.08	0.871	1.149
SNRI	−17.059	−0.23	−2.284	**0.02**	0.876	1.141
TCA	−12.469	−0.17	−1.663	0.10	0.869	1.150
Lithium	16.676	0.29	3.056	**0.00**	0.961	1.041
Anticonvulsants	12.102	0.22	2.196	**0.03**	0.887	1.127
Antipsychotics	−2.327	−0.05	−0.050	0.61	0.925	1.081
ECT	0.112	0.00	0.024	0.98	0.919	1.088

2 Dependent variable: BDNF serum levels at discharge (R = 0.32, adjusted R^2^ = 0.24, P = 0.001).

BDNF, brain-derived neurotrophic factor; ECT, electroconvulsive therapy; SNRI, serotonin and norepinephrine reuptake inhibitor; SSRI, selective serotonin reuptake inhibitor; TCA, tricyclic antidepressant; VIF, variance inflation factor.

## Discussion

The present study found higher serum BDNF levels in depressed inpatients with higher IR at the time of discharge from psychiatric hospitalization (*F* = 6.199, *P* = 0.00). This is the first report of an association between religiosity and BDNF, that, among other effects, might be consider a biological marker of neuroplasticity in patients diagnosed with depression. Furthermore, the correlation between IR and higher BDNF levels identified in the present study is consistent with previous reports showing a mainly protective effect of religiosity in depressive disorders (12). A meta-analysis of 147 studies on religion and depression involving an average of 98.975 individuals found an inverse correlation between religiosity and depression, with a larger protective effect among people under severe life stress (13). Moreover, prospective studies have confirmed the protective effect of religiosity on incident depression (3) and recovery from a depressive episode (14). In our previous study, IR was inversely correlated to suicide risk and directly correlated with resilience and quality of life of depressed inpatients (10). Taken together with the current results, this suggests BDNF may be one pathway mediating the relationship between religiosity and depression.

BDNF promotes neuronal growth, synaptic plasticity, exerts modulatory effects on gray matter in distinct subregions of the prefrontal cortex (15), and plays a key role in memory and cognition (16). In depressed patients, BDNF levels are reportedly reduced and then increases as symptoms decrease with antidepressant treatment, supporting the neurotrophic hypothesis of depressive disorders (5, 17). Increased BDNF levels were also found following short-term meditation in a 6-week randomized clinical trial in nondepressed adults (18). One hypothesis to be tested would be that religiosity might exert its protective effects against depression by increasing cortical neuroplasticity and neuroprotection through BDNF. Studies have shown that *BDNF* methylation, which decreases *BDNF* transcription, is associated with depressive-like behavior in mice (19) and depression in humans (20). Methylation of the *BDNF* promoter was also found to inversely correlate with cortical thickness in depressed individuals (20). Furthermore, an empirical study reported that a higher religiosity or spirituality was associated with greater cortical thickness in individuals at high-risk for depression (9). Hence, the epigenetic mechanism(s) of *BDNF* methylation, providing a link between environment and changes in brain neurobiology, constitutes a promising pathway for understanding how religiosity may alter adult brain function and plasticity (21).

The relationship between high IR and serum BDNF levels also provides a new route for understanding the biological effects of religiosity on the brain. Previous studies have shown an association between religious attendance and lower interleukin-6 levels, an inflammatory marker potentially related to depressive disorders (7, 8). A functional magnetic resonance imaging study of Carmelite nuns showed that recollection of spiritual, mystical experiences correlated with increased blood flow in several cortical areas, including the temporal, caudate, cingulate, orbitofrontal, and prefrontal cortices, considered to be associated with religious experiences and positive emotions (22). The results of other studies evaluating the neurobiological effect of religiosity on depressed individuals also reinforce the relevance of the current findings, reporting higher religious or spiritual importance to be a predictor of decreased default mode network connectivity, a protective biological marker in individuals at high risk for depression (9, 23).

In the present study, high IR consistently remained a statistically significant variable associated with BDNF at hospital discharge after controlling for different potential confounders, including age, sex, resilience, depressive symptoms, and tobacco consumption and psychiatric treatments. Resilience is thought to be one pathway that could explain the effects of IR in serum BDNF levels. Notably, in our study, the effects of IR on serum BDNF levels were independent of effects of resilience (*F* = 3646, *P* = 0.03). A recent meta-analysis showed the relationship between stressful life events and depression was significantly moderated by BDNF levels (24). BDNF has also been significantly associated with adaptive stress-coping strategies in a community setting (25). Alternatively, the effects of IR on BDNF levels might follow a different pathway apart from stress regulation. For example, a genetic epidemiologic study in twins reported that around 37% of religious well-being variance was explained by genetic factors (26). An increase in heritable contributions made by personal religiosity could be associated with a decreased risk of major depressive disorder, leading to distinct biological markers of depressive disorders (3).

Our conclusions must be interpreted in face of some limitations. The cross-sectional relationship between BDNF levels at hospital discharge and IR does not allow causal inferences between the variables. Multiple neurobiological and psychosocial factors, not controlled in our study, could also interfere with BDNF levels in depressive disorders in an inpatient psychiatric setting. Another set of imitations is associated with potential error rates regarding multiple statistical analysis in a reduced sample size. The results should be considered as preliminary, and replication studies are necessary for further conclusions. Nevertheless, the consistency of findings controlling for relevant potential confounders, including age, sex, resilience, depressive symptoms, tobacco consumption, and the effect of psychiatric treatments, reinforces the relationship between IR and serum BDNF levels.

## Conclusion

The present study revealed that depressed inpatients with a higher IR had higher serum BDNF levels at hospital discharge, suggesting a novel pathway to help understand the protective effects of religiosity on the brain with depressive disorders. These findings provide future approaches for investigation of the neuropsychobiological relationship between religiosity and depression.

## Data Availability

The datasets for this manuscript are not publicly available. The present study belongs to a larger cohort project in Brazil of inpatients with severe mental illness, and we would need an additional individual consent of participants to make it publicly available. Otherwise, they are readily available to editors and reviewers and to researchers upon reasonable request.

## Ethics Statement

The present project (10265) was approved at the Comite de Ética em Pesquisa, Hospital de Clínicas de Porto Alegre (HCPA). All subjects gave written informed consent and the study was carried out in accordance with the all ethical guidance and recommendations.

## Author Contributions

Study conception and design: BM, MF, and NR. Acquisition of data: BM. Analysis and interpretation of data: BM, MF, and NR. Drafting of manuscript: BM, MF, and NR. Critical revision: BM, MF, and NR.

## Funding

This study was financed in part by grants from FIPE/HCPA and by the Coordenação de Aperfeiçoamento Pessoal de Nível Superior Brasil (CAPES), Finance Code 001.

## Conflict of Interest Statement

The authors declare that the research was conducted in the absence of any commercial or financial relationships that could be construed as a potential conflict of interest.
